# Learning Oncogenetic Networks by Reducing to Mixed Integer Linear Programming

**DOI:** 10.1371/journal.pone.0065773

**Published:** 2013-06-14

**Authors:** Hossein Shahrabi Farahani, Jens Lagergren

**Affiliations:** KTH Royal Institute of Technology, Science for Life Laboratory (SciLifeLab), Center for Industrial and Applied Mathematics, School of Computer Science and Communication, Stockholm, Sweden; Shanghai Jiao Tong University School of Medicine, China

## Abstract

Cancer can be a result of accumulation of different types of genetic mutations such as copy number aberrations. The data from tumors are cross-sectional and do not contain the temporal order of the genetic events. Finding the order in which the genetic events have occurred and progression pathways are of vital importance in understanding the disease. In order to model cancer progression, we propose *Progression Networks*, a special case of Bayesian networks, that are tailored to model disease progression. Progression networks have similarities with Conjunctive Bayesian Networks (CBNs) [1],a variation of Bayesian networks also proposed for modeling disease progression. We also describe a learning algorithm for learning Bayesian networks in general and progression networks in particular. We reduce the hard problem of learning the Bayesian and progression networks to Mixed Integer Linear Programming (MILP). MILP is a Non-deterministic Polynomial-time complete (NP-complete) problem for which very good heuristics exists. We tested our algorithm on synthetic and real cytogenetic data from renal cell carcinoma. We also compared our learned progression networks with the networks proposed in earlier publications. The software is available on the website https://bitbucket.org/farahani/diprog.

## Introduction

Mutations of single nucleotides but also structural changes, e.g., deletions, duplications, inversions, and translocations of genomic segments, as well as epigentic modifications have all been implicated in cancer. Traditionally cytogenetic techniques have been used to characterize structural aberrations in tumors and more recently Comparative Genomic Hybridization (CGH) arrays have been used to reveal Copy Number Aberrations (CNA). Today, genomics resequencing, exon resequencing, and RNA-Seq are the methods of choice for assaying cancer tumors. Although this constitutes a remarkable technological development and today’s data are in many aspects unprecedented, cancer data is and will continue to be mainly cross-sectional. That is, for each of a number of patients, a single tumor is removed at one time point and assayed, which gives information about the set of aberrations in the tumor. However, aberrations occur sequentially in time and, also, one aberration can yield another aberration, by making the second favorable for the tumor (a good example is that an aberration causing angiogenesis is favorable to a tumor subsequent, but not previous, to an aberration causing increased tumor size). In fact, it is of central importance that cancer progresses and is a historical process in which the next event depends on those already having occurred. Mathematical models of cancer progression and corresponding learning algorithms are required in order to facilitate inference of cancer progression pathways, i.e., the set of favorability relations between aberrations.

Vogelstein et al. [2] suggested a path-based model of colorectal cancer progression consisting of 4 genetic events. This is a biomedical model that can be viewed as a starting point for a development of a series of mathematical models. Due to the complexity of cancer, it is desirable to base cancer models on more complex discrete structures than paths. Tree-based models are clearly more general than paths, but they do unfortunately not allow different progression paths to converge. Consequently, a substantial modeling effort has led the area to evolve from considering path-based models to tree-based models and beyond.

Desper et al. [3] suggested tree based models that are non-probabilistic, i.e., how well an individual model describe a data set cannot be assigned a probability. Beerenwinkel et al. [4] used probabilistic tree-based models and mixtures of such models. They also gave “EM-like” algorithms for learning these models. The original application was analysis of HIV data but later also cancer data was analyzed [5]. these results were subsequently improved by introduction of Hidden Variable Oncogenetic Trees (HOTs) that have a monotonicity property well-adapted to model disease progression as well as latent variables which yields a better capacity to describe noise by experimental data [6]. Global structural EM-algorithms for learning these models were also described [6].

In order to go beyond tree-based models, Hjelm et al. [7] proposed Network Aberration Models, in which aberration probabilities are based on waiting times and event histories affect waiting times in a pairwise and additive fashion. The learning algorithms for these models are straightforward heuristics that can not handle more than 12 aberrations. Beerenwinkel et al. [1], [8] proposed Conjunctive Bayesian Networks (CBNs) which is a network model with a monotonicity property. Conjunctive Bayesian networks are similar to noisy-AND models in the AI literature [9]. CBNs are not well-suited to learn noisy experimental data. Gerstung at al. [10] address this problem by proposing Hidden CBN (H-CBN) where the variables of a CBN are considered latent and visible variables have a 1-to-1 correspondence to these latent variables.

In a pioneering line of work Höglund et al. [11]–[13] used a non-probabilistic method, which incorporates Principal Component Analysis (PCA) on the pairwise correlation between aberrations and to a large extent depends on human decision and, therefore, is somewhat orthogonal to the methods described above. Cussens [14] used integer programming in pedigree reconstruction problem. Pedigrees can be seen as Bayesian networks.

Recently, by introduction of high-throughput sequencing technologies, sequencing of small collection of tumor genomes have become feasible. The temporal order of somatic mutations (point mutations and structural rearrangements) that have created a tumor genome from a germ line genome are not immediately revealed from these genomes. Greenman et al. [15] proposed an algorithm for reconstructing the events sequence of a single tumor. In a recent effort Nik-Zainal et al. [16] applied the Greenman algorithm [15] to 21 breast cancer tumors.

Gerstung and colleagues [17] observed that tumors from the same type of cancer often show a few genetic alterations in common. They hypothesized that temporal order of the events in a tumor may act on the pathway level rather than the gene level. According to their hypothesis, mutations in the genes that are involved in the same functional pathway can partly explain the heterogeneity in the tumors. After mapping the genes to the functional pathways, they applied the H-CBN algorithm on the pathway level. Cheng et al. [18] used the same approach to determine whether specific pathway alterations appear early or late during the tumor progression.

## Methods

### Notation

Learning cancer progression networks give rise to hard computational problems. In order to obtain biologically sound solutions, we need a lot of notations and mathematical apparatus. The notations and mathematical tools that are used in the paper are introduced in this section.

We use hypergraphs to represent the dependence structures of Bayesian networks. In this section, we introduce key concepts and notation for hypergraphs. We will use 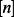 to denote the set 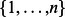. A *hypergraph* 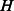 consists of a *vertex* set, denoted 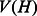, and a set of non-empty subsets of 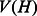 called *hyper-edges*, denoted 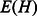. In a graph with standard meaning, all edges are 2-element subsets of 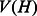. In other words, in a standard graph each edge connects two vertices. We will consider *directed hypergraphs* where each hyper-edge *e* has a unique *child*, denoted 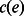, and a set of *parents*, denoted 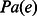. An alternative terminology would be to call 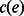 the head of *e* and 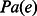 its tail. However, using the terms child and parents fits the terminology of our application better. A directed hypergraph *H* is *acyclic* if there is a linear order < on 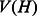 such that for every hyper-edge 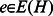 and 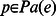, 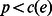. A *hyperDAG* is a *directed acyclic hypergraph*. A *k-uniform hypergraph* (*k-bounded*) is a hypergraph in which all hyper-edges contain exactly (at most) *k*-vertices. This means that a graph (with the standard meaning) is a 2-uniform hypergraph.

### The Models

We here describe the standard Bayesian network model and our models aimed to capture disease progression.

#### Bayesian and progression networks

We first introduce additional notation. We will typically consider a set of r.v.s 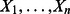. Assume 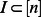. We will use 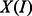 to denote the set 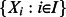. We will use 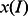 to denote assignments to 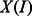, i.e., an assignment 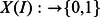. In particular, 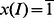 denotes that all variables in 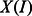 are assigned the value 1 and 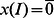 denotes that all variables in 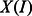 are assigned the value 0.

A *Bayesian Network* (BN) is pair 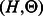, where 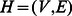 is a hyperDAG and 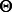 maps edges of *H* to Conditional Probability Distributions (CPDs), such that: (1) for each 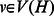, there is an associated r.v. 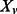 and (2) for each hyper-edge 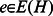, 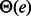 is the CPD 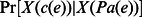. Also, we will say that a BN 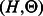 is 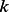 *-bounded* if the hypergraph *H* is *k*-bounded.

We now introduce our model of disease progression. A BN is called monotone if all its r.v.s are monotone, i.e., for some small constant 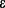 and for each hyper-edge *e*, 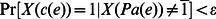. The interpretation of this model is that in order to make the child aberration advantageous to the tumor all parental aberration must have occurred. A r.v. in a BN is called *semi-monotone* if it only has non-negligible probability to be 1 in case at least one parent is 1. Formally a BN is called *semi-monotone* if all its r.v.s are semi-monotone, i.e., for some small constant 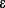 and for each hyper-edge *e*, 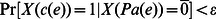. The interpretation of this model is that in order to make the child aberration advantageous to the tumor at least on parental aberration must have occurred. Monotone and semi-monotone BNs are collectively refereed to as *Progression Networks* (PNs).

**Figure 1:**
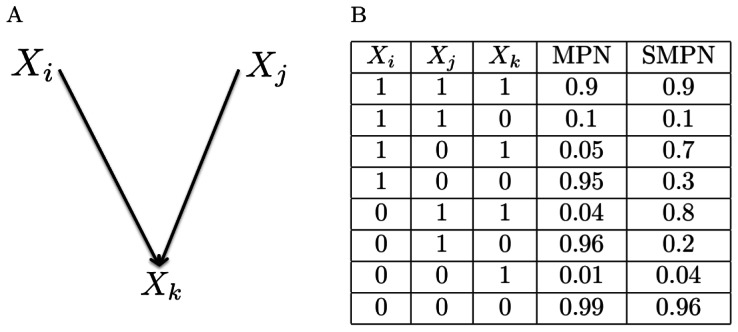
The figure shows a hyper–edge and its correspondig CPDs. A sample hyper–edge with 3 r.v.s (a) and monotone and semi–monotone CPDs for 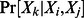 that hyper-edge (b).

Figure 1(A) shows a hyper–edge with three r.v.s 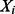, 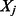, and 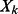. The columns that are labeled MPN and SMPN in Figure 1(B) are monotone and semi-monotone CPDs for the hyper–edge in Figure 1(A), respectively. Conjunctive Bayesian Networks that are proposed by Beerenwinkel and collaborators is a special case of our monotone BNs with 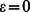, see [1].

### Learning a *k*-bounded Bayesian Network

In this section, we will show how learning a *k*-bounded BN can be reduced to the problem Maximum Weight *k*-Bounded Subhypergraph (MW*k*BS) for a weighted *k*-bounded hypergraph called the selector graph and, then, how this problem can be reduced to a Mixed Integer Linear Program (MILP). Solving a MILP problem is one, out of several NP-complete problems, for which very good heuristics have been designed. We apply the popular approach of reducing another problem to a MILP rather than developing new heuristics. We do so since by reducing the problem of learning a Bayesian network with bounded number of parents, for each random variable, to a MILP, we obtain a fast heuristic for the former problem. The algorithm can work with any decomposable score such as maximum likelihood score or Bayesian Information Criterion (BIC) score.

The reduction to MW*k*BS assigns weights in a fairly standard way, i.e., using Maximum Likelihood (ML) estimated parameters, so that the weight used is exactly what the edge contributes to the overall score of the Bayesian network. We first define weights corresponding to the ML score and, then, we show how to modify them in order to obtain weights corresponding to the BIC score. We could equivalently, as is common, use the mutual information between 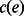 and 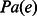 as the weight of the hyperedge *e*, see [19]. Also, since we consider decomposable scores, the weight of an edge is the same in any Bayesian network with parameters optimal for the score, which we show explicitly for the BIC score.

Assume that 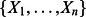 is a set of r.v.s and *Q* is a subset of it. Let *D* be a dataset and 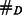 the size of the dataset. For any assignment 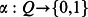, let 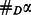 denote 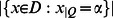 where 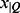 denotes the function *x* restricted to the domain *Q*. Define the *k*-bounded selector hypergraph *S* for *D* as follows. The vertex set is 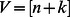, where 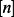 are indices corresponding to our r.v.s and 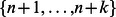 is a special set of vertices called *root parents*, which we denote *R*. The root parents are used for technical reasons as parents of vertices that otherwise would not have parents in the subDAG that finally is selected. Let *S* be obtained from the complete *k*-bounded hypergraph on 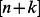 (i.e., having all possible edges) by removing all edges with a child in *R*. The weight of an hyperedge will be defined below so that it depends only on its parents that are not root parents.

Let 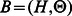 be a BN. The log-likelihood of *B*, 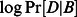, equals.
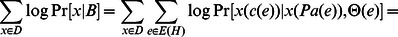


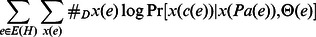



Given *H*, we define 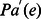 as the set of the parents of *e* that are not root parents. If 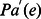 is not the empty set, the ML estimate of 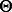 are given by
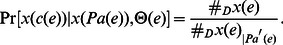
(1)


In a BN that is not monotone or semi-monotone for each edge of *H*, we define a weight 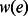 by
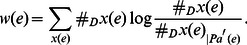
(2)


If the parent vertices in a hyper–edge *e* consist only of root parents, the weight of *e* depends only on the child vertex 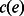. Then weight of the hyper–edge *e* can be calculated according to Equation 3

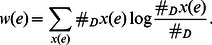
(3)


If some parents in a hyperedge *e* are root parents, instead of 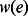 we use weight of another hyperedge 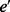 in which 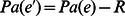 and 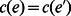. In other words when calculating the weight of a hyperedge in which some of the parents are the root parents, we ignore the root parents.

In the MPNs there is an upper bound 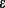 on 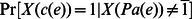. As explained before, this upper bound is applied to impose the monotonicity in the learned PN by penalizing the weight of the hyper’ edges in which the child vertex is 1 while not all parent vertices are 1. For learning MPNs we calculate all the probabilities according to Equation 1 except for the cases that 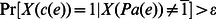 in which the weight is set to 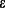. For learning SMPNs the upper bound 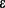 is applied to 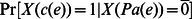.

In short, for each hyperedge a CPD like the CPD in Figure 1(B) is calculated using equation 1. In case of MPNs and SMPNs, according to their definitions, the upper bound, 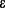, must be enforced. The values of numerator and denominator of equation 1 can be calculated from the statistics of different CNAs in the dataset according to the definition of 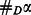 in the beginning of this section. Using the computed CPDs, then the weight of each hyperedge is calculated by equations 2 or 3.

Notice that 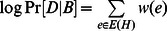. Also notice, the weight of the hyperedge *e* is independent of the rest of *H*, i.e., it is the same for any *k*-bounded hyperDAG containing *e*. We define the weight of *e* in *S* to be the weight it assumes in each of these DAGs. From this follows that, *H* is a maximum weight *k*-bounded subhyperDAG of *S* if and only if *H* together with ML estimated parameters induce a BN that maximizes the likelihood of the data.

Again, the weights that are defined above correspond to the log-likelihood or equivalently likelihood score, see [19]. When learning from noisy data using the log-likelihood score the resulting BN is typically fully connected [19]. To avoid this type of behavior Schwarz et al. [20] proposed the Bayesian Information Criterion (BIC). In contrary to the likelihood score, the BIC score provides an inclination to use simpler structures. With increasing size of the data set, however, it tends to allow more complex structures to be learned. The BIC weight of an hyperedge *e*, 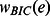, is,
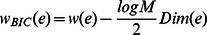
where *M* is the size of the dataset and 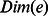 is the number of independent parameters in the hyperedge *e*.

We now describe a MILP, denoted 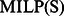, for identifying a maximum weight *k*-bounded subhyperDAG of a selector graph *S* and, thereby, complete the description of our algorithm for learning BNs. The variables of 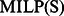 are: (1) hyperedge variables 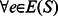, the variable 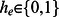 and: (2) order variables 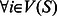, the variable 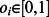.

In the formulation below, order variables facilitate the formulation of a condition that enforces the learned networks to be acyclic.

The objective function of 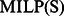 is:
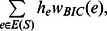
where 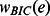 is the BIC score of *e* in the selector graph *S*.

The conditions of 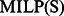 are: (1) non-root parents have exactly one incoming edge, i.e.,
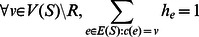
and (2) acyclic ordering of the vertices (child higher than parent), i.e.,



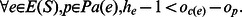



Maximizing the objective function with conditions (1) and (2) results in learning the BN with the highest score. The second condition in 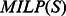 guarantees that the hypergraph induced by the program is acyclic. Because hyperedges can not contain a cycle, in each hyperedge the order variable of each parent vertex is smaller than the order variable of the child vertex. Otherwise, for at least one of the parents 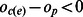 and consequently 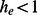. Because 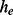 is a binary variable, 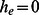. We can, hence, conclude the solution of MILP(S) will not contain a cycle.

## Results

This section contains the results of our experiments with synthetic data as well as real cytogenetic data. The synthetic data was sampled from 2 and 3-bounded BNs of all three types (i.e., MPN, SMPN, and general BNs). We learned each data set with all three variations of our algorithm.

Our main focus was the relation between aberrations. So, in order to measure the performance of our algorithm, we considered the underlying directed simple graphs of hyperDAGs in which for each parent and child of a hyper edge there is a directed edge from the parent to the child. To compare edge sets, we used the percentage of the recovered directed edges, as well as the *relative symmetric difference*, which takes both sensitivity and specificity into account. Let *M* and *L* be the set of edges in the true and the learned underlying graph, respectively. The relative symmetric difference, *F*, is defined as follows:
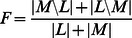
(4)


Furthermore, the values of false discovery rate 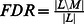 and false negative rate 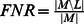 are provided separately in the supplementary material.

### Synthetic Data

We sampled data from random PNs and then tested the ability of our algorithms to learn the true models. In order to test our algorithms, we generated BNs using all possible combinations of

- 2 and 3-bounded DAGs,
- 10, 20, and 30 vertices, and
- monotone, semi-monotone, and general BNs.

Synthetic data sets were created by sampling 500, 2000, and 10000 times from each such BN. In both monotone and semi-monotone PNs 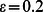. We chose the sample size 500 to show the performance of DiProg on the existing datasets, which are relatively small. The sizes of tumor datasets are constantly increasing. To measure performance of DiProg on the future larger datasets, we also tested DiProg performance on datasets with 2000 and 10000 samples.

**Figure 2:**
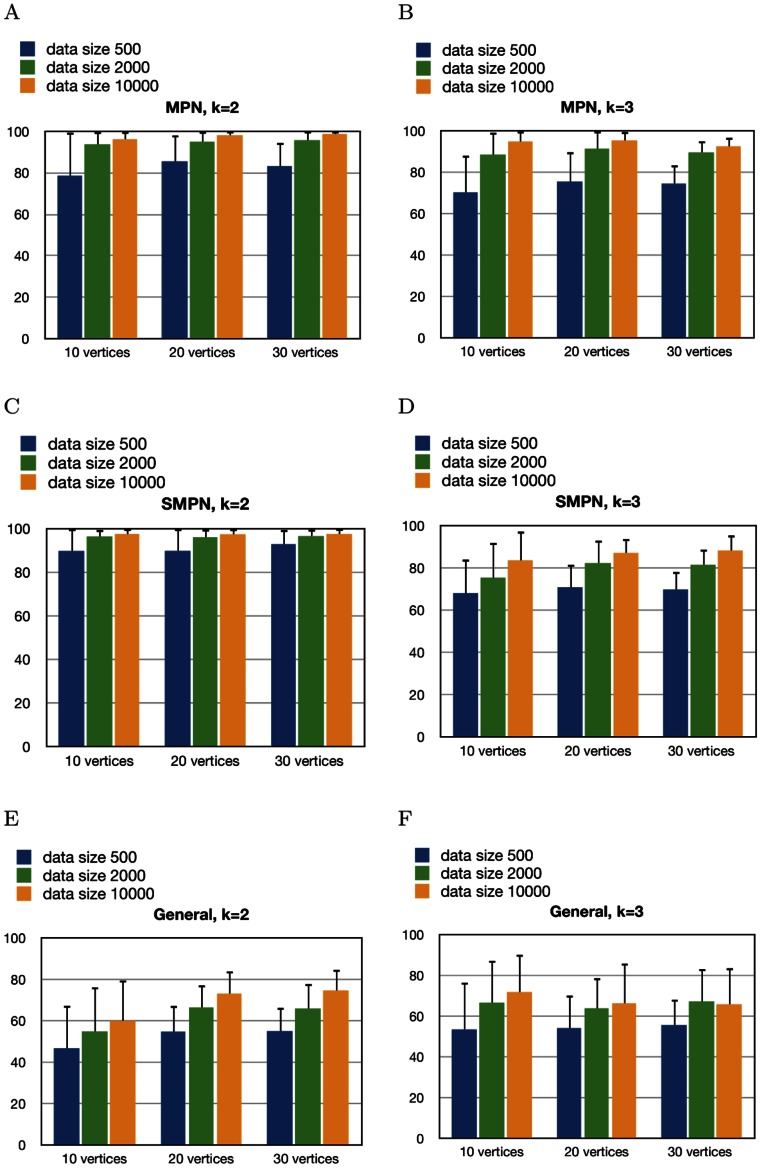
Percentage of recovered edges when data is generated with monotone, semi-monotone, and general networks and learned with the same method for 2 and 3-bounded graphs. The error bars show 1 standard deviation.

We generated 50 DAGs from each combination. The percentages of recovered edges in Figures 2(A)–2(F) are averages over 50 DAGs. Tables S1 and S2 show the percentage of the recovered edges and the relative symmetric difference when the network is learned with each of the three variations of the algorithm. Each variation of the DiProg algorithm performs best when the data is generated with the same variation. Tables S3 and S4 include the percentages of false positives and false negatives for each variation of DiProg.

All experiments were performed on a system with two quad-core Intel Harpertown 2.66 GHz CPUs (E5430) and 8 Gb of RAM, with a 64-bit Linux kernel installed. In our tests, we assigned 6 Gb of memory and a limited amount of time proportional to the number of variables and the value of *k*. The default value for maximum CPU time for each problem instance is accessible in DiProg’s help. When CPLEX did not find a provably optimal solution in the assigned time or memory constraints, we picked the best incumbent solution available in the solution pool. For each problem, DiProg receives maximum allocated time and memory as arguments.

### Cancer Data

We tested the DiProg algorithm on renal cell carcinoma (RCC) using the data from [21]. We also compared the results from DiProg algorithm with the H-CBN algorithm from [10].

#### Comparing the results with non-probabilistic methods

In [11] a dataset of 796 RCC tumors with 28 chromosomal aberrations is used. The data was retrieved from Mitelman Database of Chromosome Aberrations in Cancer [21]. To facilitate comparing our results with those in [11], we used the same dataset.

**Figure 3:**
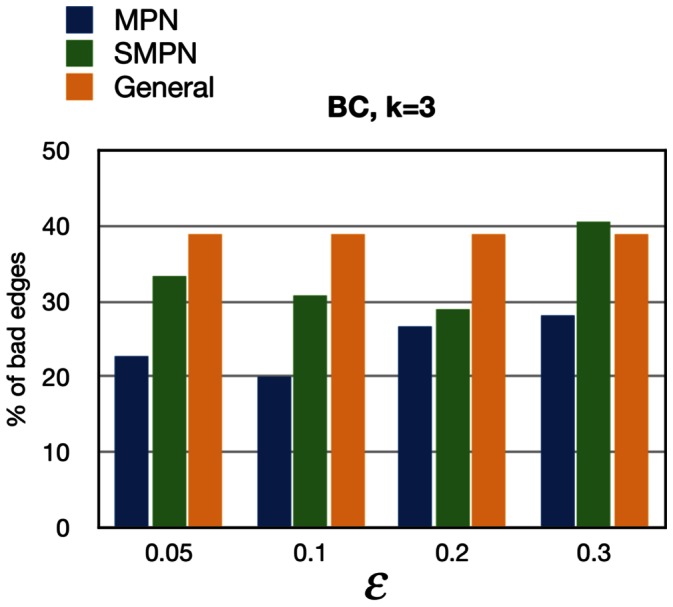
The percentage of bad edges for various values of 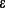 and 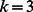 for MPN, SMPN, and General PNs for BC learned by DiProg.

The MPN and SMPN models have a free parameter, i.e., 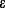. We took advantage of the breast cancer (BC) data from [13] to find a biologically realistic value of 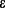 by identifying the value that gave the best correspondence between the progression network that we obtained and the network proposed for BC in [13]. This was in practice accomplished by choosing the value of 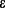 that gave the minimum fraction of bad edges relative to the number of learned edges. Following [6], we define a bad edge to be an edge in our learned PN that contradicts the partial order imposed by the progression pathways in [13]. In calculating the percentage of bad edges the edges incidents to the root parents are also counted. Figure 3 shows the percentage of bad edges in the breast cancer data from [13] with 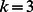 for various values of 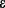. A very interesting observation is that for each value of 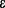 the percentage of bad edges in the learned MPNs is less than the semi-monotone and general learned PNs. Figures S1 and S2 show the percentage of bad edges in the learned BC PNs with 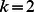 and 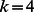 for various values of 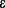. As illustrated in Figure S2, the percentage of bad edges in the learned MPN is also less than those of the learned SMPN and General, except for 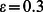. In the latter case, the large value of 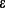 effectively weakens the monotonicity condition.

Table S5 shows the percentage of bad edges and BIC scores of the learned MPNs with different values of *k* and 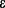. For the percentage of bad edges in the learned SMPNs and general BNs see Tables S6 and S7. For a discussion about choosing the value of 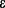 see Text S1.

**Figure 4:**
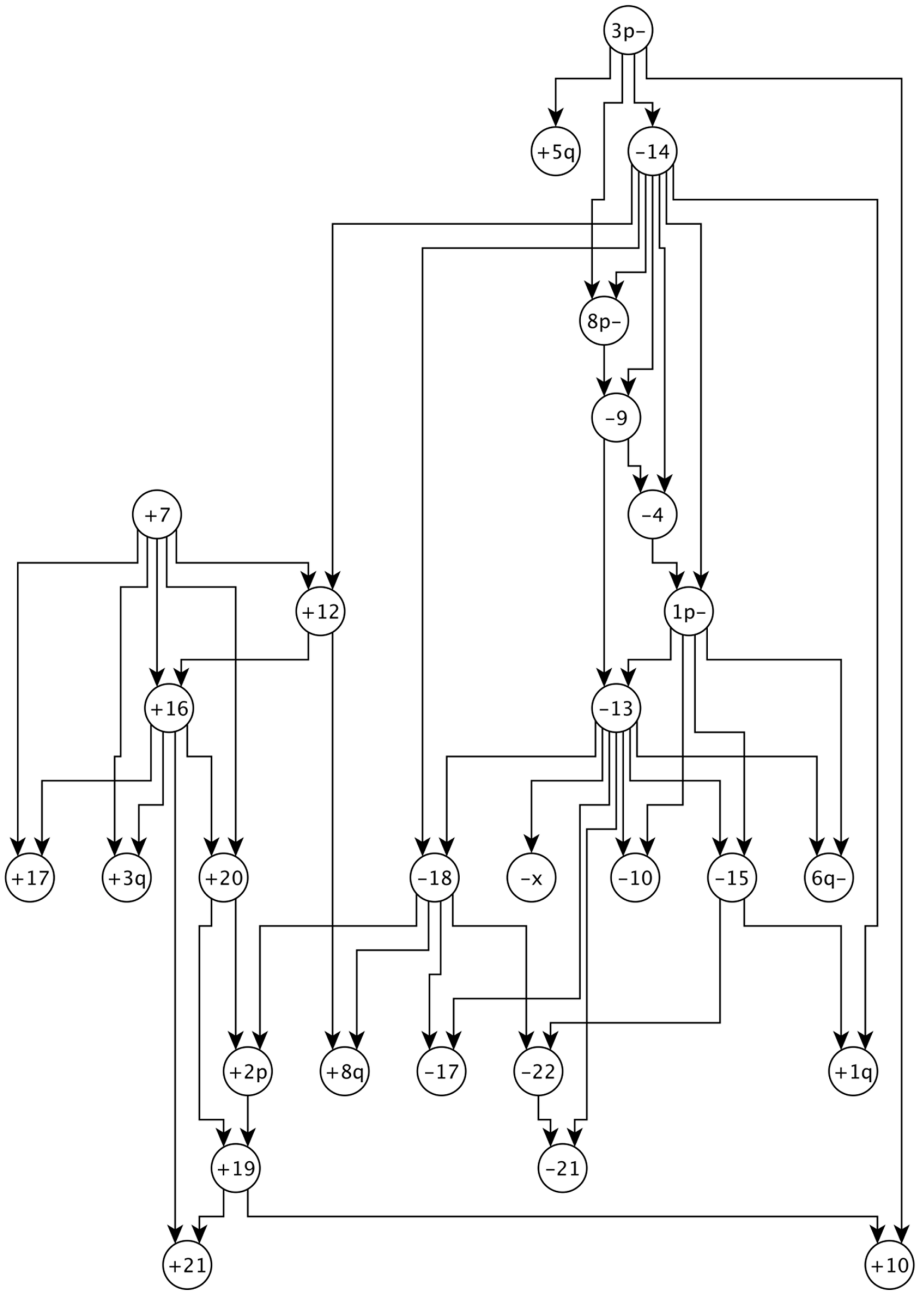
The learned MPN for RCC data with 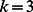 and 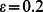. + sign stands for gain and − sign stands for loss in a chromosome arm. Long and short arms of each chromosome are denoted by *q* and *p*, respectively.

Table S8 contains the BIC scores for MPNs that are learned from RCC data in [11]. For the chosen value of 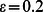, the MPN with k = 3 has the best BIC score. Figure 4 is the MPN that is learned by DiProg algorithm with 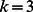 and 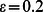 from the RCC data in [11]. The progression network for RCC that is proposed in [11] consists of two pathways, one progression pathway starts with loss 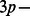 and another one starts with gains 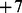 and 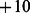 and continues with 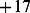, 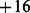, and their descendants. In our learned MPN these two pathways are distinctly separated. Most of the aberrations in the pathway in Figure 5 of [11] that starts with gains 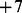 and 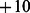 are captured in the sub-graph of our learned MPN that consists of the descendants of gain 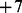. Most of the aberrations in the second pathway in [11] are captured by the component in our MPN that starts with the loss 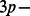 and continues with 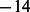 and its descendants.

**Figure 5:**
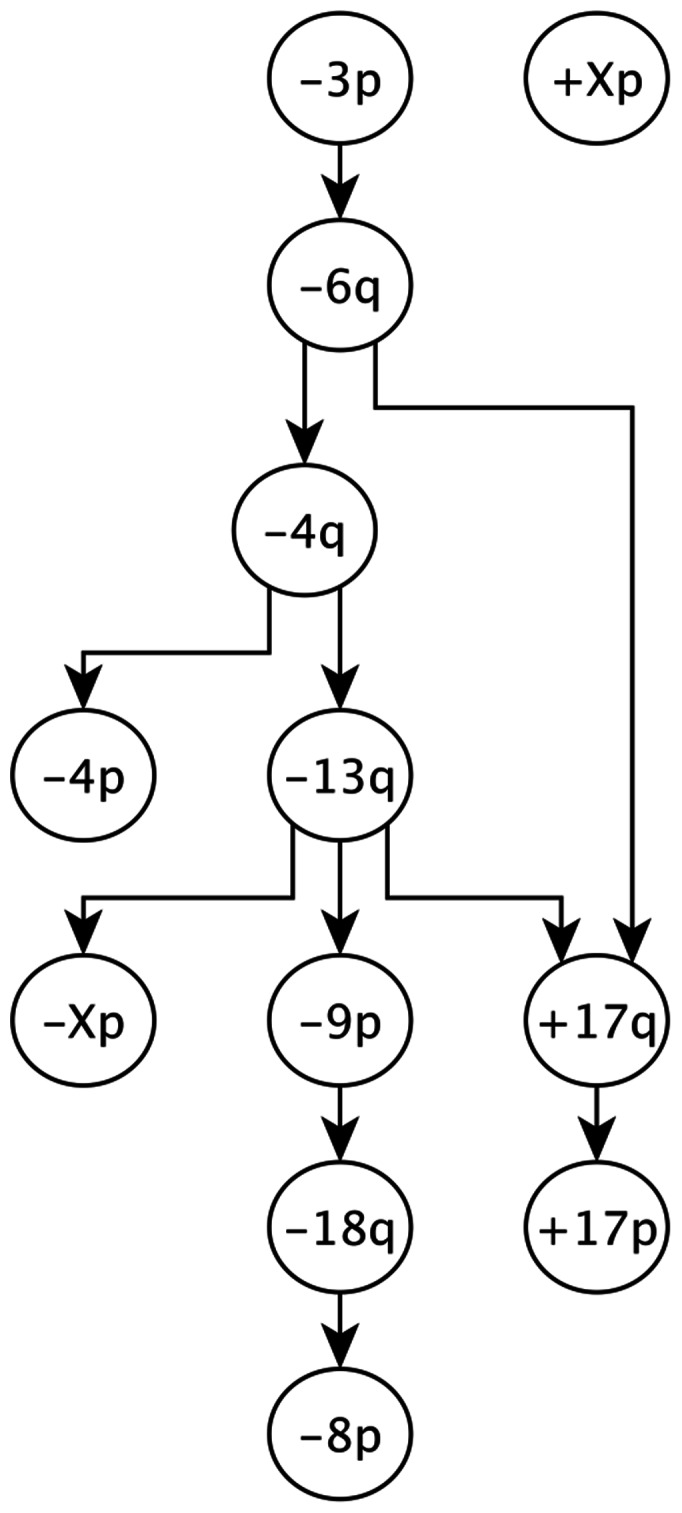
The learned MPN for RCC data with 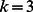 and 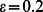. The smaller dataset in [10] is used. + sign stands for gain and − sign stands for loss in a chromosome arm. Long and short arms of each chromosome are denoted by *q* and *p*, respectively.

#### Comparison with the H-CBN algorithm

We compared the results of the DiProg with the H-CBN algorithm. Gerstung et al. [10] used RCC specific CGH data from Progenetics database [22]. Due to limitations in the number of variables that the H-CBN algorithm can handle, they restricted their analysis to 12 variables and 251 tumors in the RCC. In the previous section we presented the results of DiProg on a larger dataset from RCC with 28 variables. In order to be able to compare results of DiProg with H-CBN, in this section we applied DiProg to the smaller dataset with 12 variables. Text S2 contains a discussion about choosing the best value for *k*. Figure 5 illustrates the MPN that is learned by DiProg with 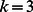 and 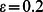. The learned MPNs by DiProg are similar to the networks that are proposed in [10] and [23]. It is noteworthy to mention that the authors in [10] rejected the network that was originally learned by the H-CBN algorithm because it only contains two edges. In order to learn a better network, they used the network proposed by Jiang et al. [23] as the starting point for a structure search by the H-CBN algorithm. So, the proposed network for RCC in [10] is sub-optimal according to the H-CBN algorithm. The network learned by DiProg is more similar to the network in [23] than the network in [10]. This shows the reliability of the DiProg results.

## Discussion

We propose new algorithms based on mixed integer linear programming (MILP) for learning BNs. Because we are especially interested in modeling disease progression, we used monotone and semi-monotone progression networks. Results from the synthetic data show that depending on the upper bound on the number of parents in monotone and semi–monotone PNs, we can learn the majority of the edges in the generating model.

Because cancer progression is a historical process, it is reasonable to assume that later aberrations need earlier aberrations to have occurred before they can be introduced to the cell. To force this monotonicity in learning the progression networks, we defined MPNs and SMPNs. In MPNs, in each hyperedge there is an upper bound on the probability of the child vertex being in the tumor if not all its parents have happened. This makes MPNs more suitable for modeling cancer progression comparing to the general BNs with no such upper bounds.

We also applied our algorithm to cytogenetic data from [11]. In contrast to the semi-automatic method by Höglund et al., DiProg is automatic and,therefore, needs less support by human decision. Furthermore DiProg is based on probabilistic models, which can generate synthetic data and assign a likelihood to the biological data. Also comparing our algorithm with the H-CBN algorithm [10] on the same smaller dataset from RCC shows that DiProg results are in agreement with the previously published results. Comparing to the H-CBN algorithm, DiProg can handle substantially more variables. To illustrate this we presented the results of DiProg on synthetic data with 30 variables and on cytogenetic data from RCC with 28 variables.

## Supporting Information

Figure S1
**The percentage of bad edges for various values of 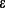 and 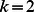 for MPN/SMPN, and General PNs for BC learned by DiProg.**
(EPS)

Figure S2
**The percentage of bad edges for various values of 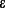 and 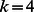 for MPN, SMPN, and General PNs for BC learned by DiProg.**
(EPS)

Table S1
**Performance of the algorithm with synthetic data for 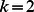.**
(PDF)

Table S2
**Performance of the algorithm with synthetic data for 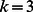.**
(PDF)

Table S3
**False discovery rate and false negative rate values for synthetic data with 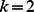.**
(PDF)

Table S4
**False discovery rate and false negative rate values for synthetic data with 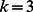.**
(PDF)

Table S5 **Percentage of bad edges and the BIC scores of the MPNs learned from the BC data in** [**13**] **with DiProg.**
(PDF)

Table S6 **Percentage of bad edges and the BIC scores of the SMPNs learned from the BC data in** [**13**] **with DiProg algorithm.**
(PDF)

Table S7 **Percentage of bad edges and the BIC scores of the General BNs learned from the BC data in** [**13**] **with DiProg algorithm.**
(PDF)

Table S8
**The BIC scores of the MPNs learned from the RCC data in** [**11**] **with DiProg algorithm.** As explained in Text S1 of the supplementary material the best value for 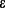 that gives the biologically sound PNs is 0.2. For 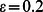 the learned MPN with 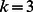 has the largest BIC score.(PDF)

Text S1
**Choosing the value of 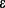.**
(PDF)

Text S2 **The BIC scores of the MPNs learned from the RCC data in** [**10**] **with DiProg algorithm.**
(PDF)
