# Supplementary material for: Learning Oncogenetic Networks by Reducing to Mixed Integer Linear Programming
Source: PLoS One. 2013 Jun 14;8(6):e65773. doi: 10.1371/journal.pone.0065773 (PMC3683041; doi:10.1371/journal.pone.0065773)
Supplement: Table S1 — Performance of the algorithm with synthetic data for . (PDF) [file pone.0065773.s003.pdf]

$\mathbf{V}^a$	$\mathbf{S}^b$	**Generator**[c]	**MPN**[d]	**SMPN** [e]	**General**[f]	${F_{MPN}}^g$	${F_{SMPN}}^h$	${F_{General}}^i$
10	500	General	23.36	23.36	46.68	69.12	69.12	48
10	500	MPN	78.73	78.73	59.27	20.01	20.01	40.6
10	500	SMPN	89.87	89.87	66.5	9.14	9.14	33.72
10	2000	General	26.65	26.65	54.93	65.77	65.77	41.76
10	2000	MPN	93.76	93.76	70.47	5.59	5.59	29.58
10	2000	SMPN	96.52	96.52	66.3	3.21	3.21	33.84
10	10000	General	26.62	26.62	60.05	66.31	66.31	38.25
10	10000	MPN	96.26	96.26	70.35	3.74	3.74	29.85
10	10000	SMPN	97.59	97.59	73.28	2.41	2.41	27.01
20	500	General	23.08	23.08	54.78	69.46	69.46	41.98
20	500	MPN	85.61	85.38	65.74	13.23	13.46	34.72
20	500	SMPN	89.9	89.9	73.29	8.84	8.84	26.99
20	2000	General	25.85	25.85	66.47	66.55	66.55	31.04
20	2000	MPN	95.1	95.1	73.63	4.55	4.55	26.64
20	2000	SMPN	96.15	96.15	76.74	3.31	3.31	23.52
20	10000	General	26.28	26.28	73.23	66	66	25.57
20	10000	MPN	98.22	98.22	75.86	1.72	1.72	24.3
20	10000	SMPN	97.51	97.51	79.64	2.39	2.39	20.82
30	500	General	20.96	20.96	55.04	71.53	71.53	42.21
30	500	MPN	83.25	82.57	70.5	15.65	16.36	30.39
30	500	SMPN	92.99	92.99	75.23	6.07	6.07	26.55
30	2000	General	24.6	24.6	65.95	67.18	67.18	31.73
30	2000	MPN	95.82	95.58	78.28	3.79	3.99	22.4
30	2000	SMPN	96.66	96.66	80.62	3.02	3.02	20.15
30	10000	General	25.92	25.92	74.66	65.75	65.75	23.87
30	10000	MPN	98.72	98.72	78.04	1.28	1.28	22.57
30	10000	SMPN	97.64	97.64	81.51	2.21	2.21	18.84

Table S 1: Performance of the algorithm with synthetic data for k=2

[a] Vertex number
[b] Sample size
[c] Type of generator
[d] Percentage of learned edges with MPN variation of the alagorithm
[e] Percentage of learned edges with SMPN variation of the algorithm
[f] Percentage of learned edges with General variation of the algorithm
[g] Value of parameter F for the MPN variation of the algorithm
[h] Value of parameter F for the SMPN variation of the algorithm
[i] Value of parameter F for the General variation of the algorithm
