# Supplementary material for: Learning Oncogenetic Networks by Reducing to Mixed Integer Linear Programming
Source: PLoS One. 2013 Jun 14;8(6):e65773. doi: 10.1371/journal.pone.0065773 (PMC3683041; doi:10.1371/journal.pone.0065773)
Supplement: Table S2 — Performance of the algorithm with synthetic data for . (PDF) [file pone.0065773.s004.pdf]

**V**[a]	**S**[b]	**Generator**[c]	**MPN**[d]	**SMPN**[e]	**General**[f]	F_{MPN}[g]	F_{SMPN}[h]	$F_{General}$[i]
10	500	General	21.95	32.85	53.55	70.76	64.06	41.33
10	500	MPN	70.38	69.06	46.91	23.35	25.71	50.97
10	500	SMPN	64.34	68.09	60.04	29.14	33.16	38.19
10	2000	General	23.82	40.11	66.64	69.38	60.28	30.1
10	2000	MPN	88.54	84.55	60.45	8.19	13.24	37.89
10	2000	SMPN	77.5	75.36	69.68	19.15	30.06	29.13
10	10000	General	24.44	42.2	71.9	69.27	60.08	26.05
10	10000	MPN	94.94	92.75	72.97	4.62	7.21	26.68
10	10000	SMPN	82.57	83.65	75.06	15.41	23.81	24.43
20	500	General	20.33	35.09	54.19	72.1	62.11	41.32
20	500	MPN	75.54	64.17	59.87	19.83	35.09	39.06
20	500	SMPN	65.68	70.88	66.92	27.38	31.79	33.03
20	2000	General	22.94	44.32	63.94	69.5	56.6	33.23
20	2000	MPN	91.45	78.97	72.6	6.6	23.07	28.96
20	2000	SMPN	78.03	82.31	76.55	18.17	23.53	24.74
20	10000	General	24.53	50.61	66.36	68.3	52.87	33.57
20	10000	MPN	95.36	86.41	81.1	4.25	16.4	20.71
20	10000	SMPN	85.47	87.13	82.36	12.37	20.36	19.73
30	500	General	21.69	39.83	55.71	70.24	58.1	41.13
30	500	MPN	74.53	61.88	60.65	22.24	39.4	40.68
30	500	SMPN	69.56	69.8	67.3	34.01	23.72	34.25
30	2000	General	22.99	46.13	67.24	69.53	55.19	31.63
30	2000	MPN	89.64	73.31	71.29	9.69	30.91	32.65
30	2000	SMPN	77.24	81.57	74	28.88	15.05	29.29
30	10000	General	24.96	50.31	65.87	67.59	52.72	35.64
30	10000	MPN	92.51	73.59	70.59	7.82	32.03	34.81
30	10000	SMPN	76.04	88.29	76.38	31.09	10.32	27.61

Table S 2: Performance of the algorithm with synthetic data for k=3

[a] Vertex number
[b] Sample size
[c] Type of generator
[d] Percentage of learned edges with MPN variation of the alagorithm
[e] Percentage of learned edges with SMPN variation of the algorithm
[f] Percentage of learned edges with General variation of the algorithm
[g] Value of parameter F for the MPN variation of the algorithm
[h] Value of parameter F for the SMPN variation of the algorithm
[i] Value of parameter F for the General variation of the algorithm
