# Supplementary material for: Learning Oncogenetic Networks by Reducing to Mixed Integer Linear Programming
Source: PLoS One. 2013 Jun 14;8(6):e65773. doi: 10.1371/journal.pone.0065773 (PMC3683041; doi:10.1371/journal.pone.0065773)
Supplement: Table S3 — False discovery rate and false negative rate values for synthetic data with . (PDF) [file pone.0065773.s005.pdf]

$\mathbf{V}^{a}$	$\mathbf{S}^{b}$	**Generator** [c]	MPN_{FDR} [d]	MPN_{FNR} [e]	$SMPN_{FDR}$ [f]	$SMPN_{FNR}$ [g]	G_{FDR} [h]	G_{FNR} [i]
10	500	General	46.73	76.64	46.73	76.64	39.11	53.32
10	500	MPN	17.31	21.27	17.31	21.27	39.98	40.73
10	500	SMPN	7.53	10.13	7.53	10.13	33.7	33.5
10	2000	General	47.5	73.35	47.5	73.35	36.97	45.07
10	2000	MPN	4.2	6.24	4.2	6.24	29.56	29.53
10	2000	SMPN	2.81	3.48	2.81	3.48	33.84	33.7
10	10000	General	51.67	73.38	51.67	73.38	36.08	39.95
10	10000	MPN	3.74	3.74	3.74	3.74	30.02	29.65
10	10000	SMPN	2.41	2.41	2.41	2.41	27.26	26.72
20	500	General	53.4	76.92	53.4	76.92	37.8	45.22
20	500	MPN	11.87	14.39	12.1	14.62	33.06	34.26
20	500	SMPN	7.23	10.1	7.23	10.1	27.11	26.71
20	2000	General	51.16	74.15	51.16	74.15	27.98	33.53
20	2000	MPN	4.14	4.9	4.14	4.9	26.81	26.37
20	2000	SMPN	2.63	3.85	2.63	3.85	23.68	23.26
20	10000	General	50.34	73.72	50.34	73.72	24.21	26.77
20	10000	MPN	1.66	1.78	1.66	1.78	24.45	24.14
20	10000	SMPN	2.28	2.49	2.28	2.49	21.23	20.36
30	500	General	54.91	79.04	54.91	79.04	38.81	44.96
30	500	MPN	14.4	16.75	13.14	17.43	31.16	29.5
30	500	SMPN	5	7.01	5	7.01	28.18	24.77
30	2000	General	50.07	75.4	50.07	75.4	28.95	34.05
30	2000	MPN	3.35	4.18	3.52	4.42	23.04	21.72
30	2000	SMPN	2.68	3.34	2.68	3.34	20.84	19.38
30	10000	General	48.73	74.08	48.73	74.08	22.26	25.34
30	10000	MPN	1.28	1.28	1.28	1.28	23.14	21.96
30	10000	SMPN	2.05	2.36	2.05	2.36	19.16	18.49

Table S 3: False positive and false negative values for synthetic data with $k = 2$

[a] Vertex number
[b] Sample size
[c] Type of generator
[d] Value of FDR from the MPN variation of DiProg
[e] Value of FNR from the MPN variation of DiProg
[f] Value of FDR from the SMPN variation of DiProg
[g] Value of FNR from the SMPN variation of DiProg
[h] Value of FDR from the General variation of DiProg
[i] Value of FNR from the General variation of DiProg
