# Supplementary material for: Learning Oncogenetic Networks by Reducing to Mixed Integer Linear Programming
Source: PLoS One. 2013 Jun 14;8(6):e65773. doi: 10.1371/journal.pone.0065773 (PMC3683041; doi:10.1371/journal.pone.0065773)
Supplement: Table S4 — False discovery rate and false negative rate values for synthetic data with . (PDF) [file pone.0065773.s006.pdf]

$\mathbf{V}^{a}$	$\mathbf{S}^{b}$	**Generator** [c]	MPN_{FDR} [d]	MPN_{FNR} [e]	$SMPN_{FDR}$ [f]	$SMPN_{FNR}$ [g]	G_{FDR} [h]	G_{FNR} [i]
10	500	General	51.2	78.05	58.97	67.15	33.91	46.45
10	500	MPN	14.72	29.62	18.33	30.94	47.83	53.09
10	500	SMPN	20.09	35.66	33.83	31.91	35.74	39.96
10	2000	General	55.54	76.18	59.47	59.89	24.4	33.36
10	2000	MPN	4.05	11.46	10.29	15.45	31.31	39.55
10	2000	SMPN	14.48	22.5	34.41	24.64	24.55	30.32
10	10000	General	57.15	75.56	61.32	57.8	22.77	28.1
10	10000	MPN	4.05	5.06	7.05	7.25	25.58	27.03
10	10000	SMPN	13.05	17.43	29.71	16.35	21.51	24.94
20	500	General	53.15	79.67	54.1	64.91	31.33	45.81
20	500	MPN	14.07	24.46	33.87	35.83	37.61	40.13
20	500	SMPN	18.37	34.32	34.04	29.12	32.79	33.08
20	2000	General	52.79	77.06	55.05	55.68	27.85	36.06
20	2000	MPN	4.38	8.55	24.81	21.03	30.3	27.4
20	2000	SMPN	13.69	21.97	28.45	17.69	25.87	23.45
20	10000	General	53.63	75.47	55.53	49.39	29.29	33.64
20	10000	MPN	3.82	4.64	18.86	13.59	22.32	18.9
20	10000	SMPN	9.92	14.53	26.49	12.87	21.62	17.64
30	500	General	49.36	78.31	55.35	60.17	35.28	44.29
30	500	MPN	18.57	25.47	40.47	38.12	41.78	39.35
30	500	SMPN	37.1	30.44	15.73	30.2	35.6	32.7
30	2000	General	53.99	77.01	56.25	53.87	28.2	32.76
30	2000	MPN	8.97	10.36	34.51	26.69	36.02	28.71
30	2000	SMPN	34.01	22.76	11.24	18.43	32.21	26
30	10000	General	52.74	75.04	55.19	49.69	32.97	34.13
30	10000	MPN	8.11	7.49	36.72	26.41	39.35	29.41
30	10000	SMPN	36.9	23.96	8.82	11.71	31.04	23.62

Table S 4: False positive and false negative values for synthetic data with $k = 3$

[a] Vertex number
[b] Sample size
[c] Type of generator
[d] Value of FDR from the MPN variation of DiProg
[e] Value of FNR from the MPN variation of DiProg
[f] Value of FDR from the SMPN variation of DiProg
[g] Value of FNR from the SMPN variation of DiProg
[h] Value of FDR from the General variation of DiProg
[i] Value of FNR from the General variation of DiProg
