# Supplementary material for: Learning Oncogenetic Networks by Reducing to Mixed Integer Linear Programming
Source: PLoS One. 2013 Jun 14;8(6):e65773. doi: 10.1371/journal.pone.0065773 (PMC3683041; doi:10.1371/journal.pone.0065773)
Supplement: Table S5 — Percentage of bad edges and the BIC scores of the MPNs learned from the BC data in [13] with DiProg. (PDF) [file pone.0065773.s007.pdf]

k [a]	ε [b]	**BE%** [c]	**BIC score** [d]
2	0.05	22.727	-5398.130
2	0.10	22.727	-5269.224
2	0.20	18.182	-5200.271
2	0.30	22.727	-5197.827
3	0.05	22.727	-5398.130
3	0.10	20.000	-5264.613
3	0.20	30.000	-5154.744
3	0.30	30.303	-5135.192
4	0.05	22.727	-5398.130
4	0.10	20.000	-5264.613
4	0.20	20.690	-5168.981
4	0.30	39.394	-5139.866

Table S 5: Percentage of bad edges and the BIC scores of the MPNs learned from the BC data in [13] with DiProg

[a] Maximum number of vertices in each hyperedge
[b] Value of ε in MPN learning
[c] Percentage of bad edges.
[d] The BIC score of the learned MPN by DiProg.
