# Supplementary material for: Learning Oncogenetic Networks by Reducing to Mixed Integer Linear Programming
Source: PLoS One. 2013 Jun 14;8(6):e65773. doi: 10.1371/journal.pone.0065773 (PMC3683041; doi:10.1371/journal.pone.0065773)
Supplement: Table S6 — Percentage of bad edges and the BIC scores of the SMPNs learned from the BC data in [13] with DiProg algorithm. (PDF) [file pone.0065773.s008.pdf]

| $k$ [a] | $\varepsilon$ [b] | **BE%** [c] | **BIC score** [d] |
|---|---|---|---|
| 3 | 0.05 | 36.111 | -5213.594 |
| 3 | 0.10 | 29.730 | -5147.824 |
| 3 | 0.20 | 34.211 | -5120.994 |
| 3 | 0.30 | 35.135 | -5120.670 |
| 4 | 0.05 | 30.000 | -5215.807 |
| 4 | 0.10 | 32.500 | -5155.409 |
| 4 | 0.20 | 38.462 | -5125.019 |
| 4 | 0.30 | 39.024 | -5120.280 |

Table S 6: Percentage of bad edges and the BIC scores of the SMPNs learned from the BC data in [13] with DiProg algorithm

[a] Maximum number of vertices in each hyperedge
[b] Value of $\varepsilon$ in SMPN learning
[c] Percentage of bad edges.
[d] The BIC score of the learned SMPN by DiProg.
