# Supplementary material for: Learning Oncogenetic Networks by Reducing to Mixed Integer Linear Programming
Source: PLoS One. 2013 Jun 14;8(6):e65773. doi: 10.1371/journal.pone.0065773 (PMC3683041; doi:10.1371/journal.pone.0065773)
Supplement: Table S7 — Percentage of bad edges and the BIC scores of the General BNs learned from the BC data in [13] with DiProg algorithm. (PDF) [file pone.0065773.s009.pdf]

k [a]	ε [b]	**BE%** [c]	**BIC score** [d]
2	NA	22.727	-5197.827
3	NA	32.432	-5120.670
4	NA	40.000	-5126.657

Table S 7: Percentage of bad edges and the BIC scores of the General BNs learned from the BC data in [13] with DiProg algorithm

[a] Maximum number of vertices in each hyperedge
[b] Value of ε
[c] Percentage of bad edges.
[d] The BIC score of the learned General BNs by DiProg.
