# Supplementary material for: Learning Oncogenetic Networks by Reducing to Mixed Integer Linear Programming
Source: PLoS One. 2013 Jun 14;8(6):e65773. doi: 10.1371/journal.pone.0065773 (PMC3683041; doi:10.1371/journal.pone.0065773)
Supplement: Table S8 — The BIC scores of the MPNs learned from the RCC data in [11] with DiProg algorithm. As explained in Text S1 of the supplementary material the best value for that gives the biologically sound PNs is 0.2. For the learned MPN with has the largest BIC score. (PDF) [file pone.0065773.s010.pdf]

k [a]	ε [b]	**BIC score** [c]
2	0.05	-7869.314
2	0.1	-7767.491
2	0.2	-7759.997
2	0.3	-7729.563
3	0.05	-7868.416
3	0.1	-7735.850
3	0.2	-7620.549
3	0.3	-7562.661
4	0.05	-7868.416
4	0.1	-7735.850
4	0.2	-7687.373
4	0.3	-7623.140

Table S 8: The BIC scores of the MPNs learned from the RCC data in [11] with DiProg algorithm. As explained in Text S1 of the supplementary material the best value for ε that gives the biologically sound PNs is 0.2. For $\varepsilon = 0.2$ the learned MPN with $k = 3$ has the largest BIC score.

[a] Maximum number of vertices in hyperedges
[b] Value of ε
[c] The BIC score of the learned General BNs by DiProg.
