# Supplementary material for: Learning Oncogenetic Networks by Reducing to Mixed Integer Linear Programming
Source: PLoS One. 2013 Jun 14;8(6):e65773. doi: 10.1371/journal.pone.0065773 (PMC3683041; doi:10.1371/journal.pone.0065773)
Supplement: Text S1 — Choosing the value of . (PDF) [file pone.0065773.s011.pdf]

Text S1. Choosing the value of ε

There is an important fact to take into consideration before choosing the best value of ε, that is, for the same type of the PN and the same value of ε, if the MILP reaches the optimal solution the BIC score of a learned PN with a larger k must be at least as large as the BIC score of the learned PN with a smaller k. As explained in the paper, if CPLEX does not find an optimal solution before reaching the maximum allocated time or memory, we pick the best incumbent solution from the solution pool. For example, if for $k = 4$ and some value of ε, CPLEX returns a solution with less BIC score than the solution for $k = 3$ and the same value of ε, we conclude that the latter solution is a suboptimal solution comparing to the former.
According to the Supplementary Table S5, the MPN that is learned with $\varepsilon = 0.2$ and $k = 2$ has the minimum fraction of bad edges. So we pick $\varepsilon = 0.2$ as the value for ε that gives the best biologically sound MPN.
