# Supplementary material for: Learning Oncogenetic Networks by Reducing to Mixed Integer Linear Programming
Source: PLoS One. 2013 Jun 14;8(6):e65773. doi: 10.1371/journal.pone.0065773 (PMC3683041; doi:10.1371/journal.pone.0065773)
Supplement: Text S2 — The BIC scores of the MPNs learned from the RCC data in [10] with DiProg algorithm. (PDF) [file pone.0065773.s012.pdf]

Text S2. Comparing the DiProg with the H-CBN algorithm

Supplementary Table S9 contains the BIC scores for the MPNs that are learned by the DiProg from the RCC data in [10]. Again, we compare the BIC scores of the learned MPNs with $\varepsilon = 0.2$. The BIC scores for $k = 3$ and $k = 4$ are equal and both larger than the BIC score for $k = 2$. An inspection of the learned MPNs showed that the structures of the learned MPNs with $k = 3$ and $k = 4$ are exactly similar. We pick the learned MPN corresponding to $\varepsilon = 0.2$ and $k = 3$ or 4 as the best model. Figure 5 in the paper shows the learned MPN with the chosen parameters.

k [a]	ε [b]	**BIC score** [c]
2	0.05	-1352.261
2	0.1	-1317.717
2	0.2	-1308.193
2	0.3	-1308.193
3	0.05	-1352.261
3	0.1	-1317.717
3	0.2	-1306.452
3	0.3	-1303.916
4	0.05	-1352.261
4	0.1	-1317.717
4	0.2	-1306.452
4	0.3	-1303.916

Table S 9: The BIC scores of the MPNs learned from the RCC data in [10] with DiProg algorithm

[a] Maximum number of vertices in hyperedges
[b] Value of ε
[c] The BIC score of the learned General BNs by DiProg.
